# Deconvoluting cellular neighborhoods in pancreatic ductal adenocarcinoma

**DOI:** 10.1038/s42003-022-04032-1

**Published:** 2022-10-17

**Authors:** Jessica Castrillon Lal

**Affiliations:** 1 Genomic Medicine Institute, Lerner Research Institute, Cleveland Clinic, Cleveland, OH 44195 USA; 2 Department of Molecular Medicine, Cleveland Clinic Lerner College of Medicine, Case Western Reserve University, Cleveland, OH 44195 USA

## Abstract

Pancreatic ductal adenocarcinoma (PDAC) is one of the most aggressive cancers, with less than 10% of patients surviving more than five years. A major challenge of PDAC is understanding the complexity of the tumor architecture and identifying targetable cellular phenotypes that can inform clinical decision-making. Hwang et al. recently harnessed spatially-resolved transcriptomics to characterize gene expression profiles across PDAC tumor slices and identified recurrent expression patterns in malignant and fibroblast cellular neighborhoods that correlated with treatment outcomes.

Pancreatic Ductal Adenocarcinoma (PDAC) is the most common form of pancreatic cancer and among the leading causes of cancer deaths. Long-lasting treatment options for PDAC are primarily limited to surgery followed by adjuvant chemotherapy, a blunt-force method that targets highly-proliferative cells, though most patients still succumb to the disease. In order to improve treatment strategies for PDAC, it is first essential to understand how cancer cells evolve and populate neighboring tissues. The tumor microenvironment plays an essential role in tumor progression, as cells are sensitive and quickly respond to changes in local cues. Single-nucleus RNA sequencing and digital spatial profiling techniques allow researchers to understand the complexity of the tumor microenvironment with higher granularity by providing gene expression data and spatial distribution of individual cells. While spatial transcriptomics is a powerful tool that maps cellular location and function, the addition of single-cell gene expression data can further improve our understanding of rapid and dynamic expression patterns relevant to PDAC progression.

Hwang et al. recently utilized single-nucleus RNA sequencing and digital spatial profiling in PDAC tumors and organoids to help clarify the differences in cellular composition between chemoradiation-treated and control (untreated) tumor samples^1^. While the standard cellular profiles of PDAC are classic/epithelial cellular phenotypes and basal-like/squamous, mesenchymal states, the authors identified additional gene signatures and cancer-associated fibroblasts that might explain tumor evolution and progression. The authors uncovered malignant neural-like progenitor cellular gene signatures that were enriched in the chemoradiation-treated tumors. Altogether, the authors defined four unique cancer-associated fibroblast cell states: myofibroblasts, neurotropic, immunomodulatory, and adhesive gene signatures. Notably, an increase in the adhesive cancer-associated fibroblast signature was associated with poor survival in the treatment group, while the myofibroblast cancer-associated fibroblast signature was decreased in the treatment group. Three-dimensional spatial mapping of these gene expression profiles also identified three major cellular clusters that illustrated the complexity of PDAC tumors.

Altogether, this study harnessed a spatial transcriptomic dataset in PDAC to better understand the tumor microenvironment which can infer cellular interactions that might indicate potential druggable targets. For example, the authors identified increased expression of the receptor-ligand pair, *CXCL12-CXCR4*, in the chemoradiation group. *CXCL12-CXCR4* is highly expressed in the cancer cell and immune cell interactions, suggesting that blocking this interaction could benefit chemoradiation-treated PDAC patients. Although single-cell transcriptomics can help resolve challenges in understanding cancer invasion and progression, additional standard data pipelines are needed to optimize cellular segmentation and reliably annotate cell types based on these transcriptomic profiles. The evolution of this technology will facilitate tissue-scale resolution of cancer biopsies and could ultimately impact precision-medicine drug discovery in challenging diseases like PDAC.

**Figure:**
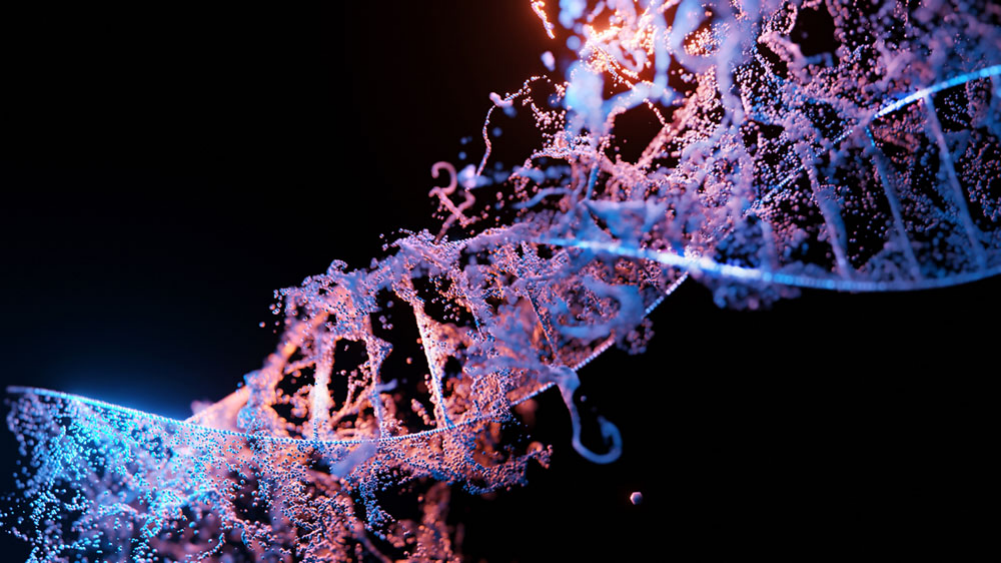
Sangharsh Lohakare
